# Supplementary figures and images for: RoPod, a customizable toolkit for non-invasive root imaging, reveals cell type-specific dynamics of plant autophagy
Source: Sci Rep. 2024 Jun 3;14:12664. doi: 10.1038/s41598-024-63226-1 (PMC11148066; doi:10.1038/s41598-024-63226-1)

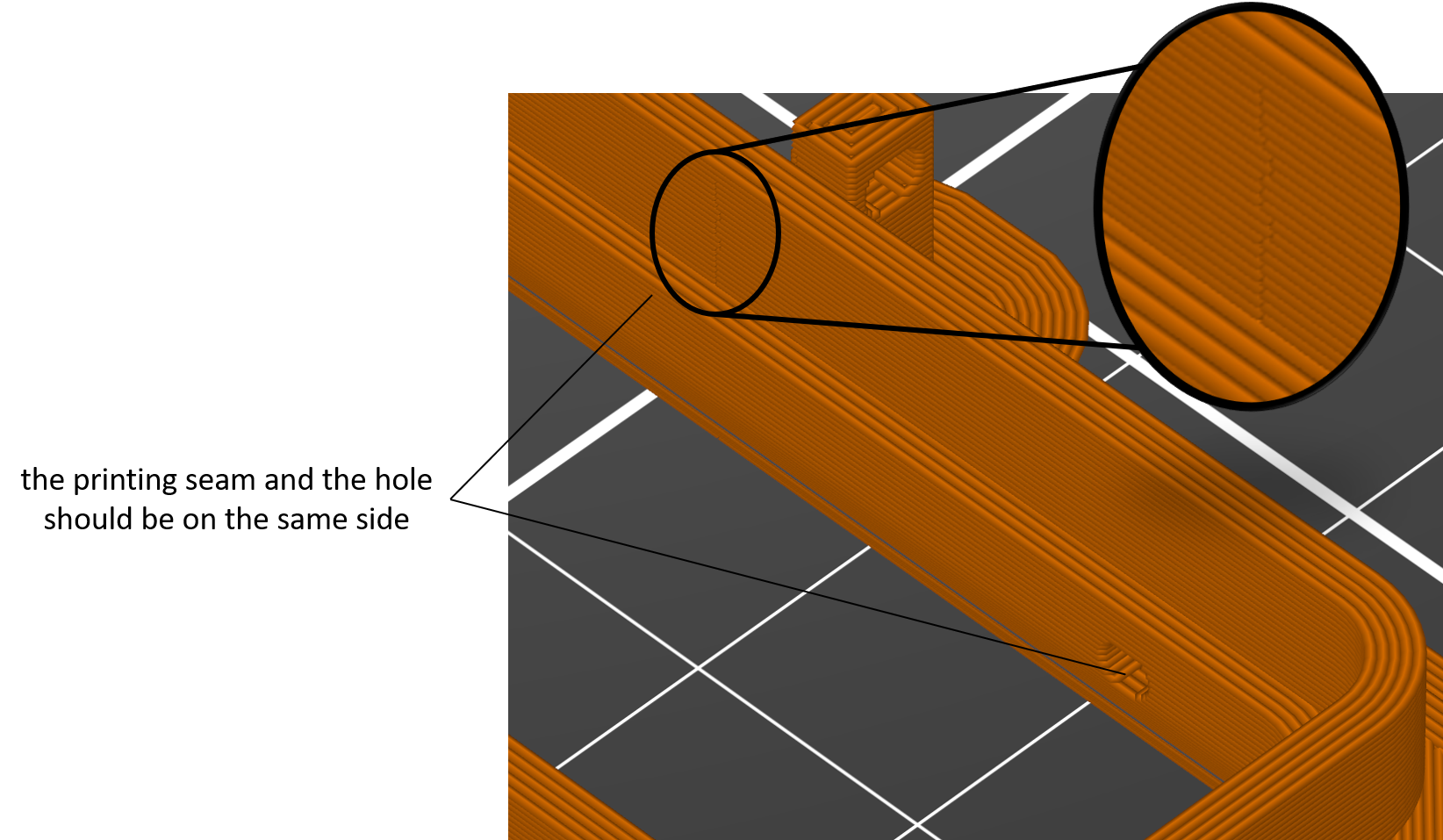

Supplement: Supplementary file 6 — Supplementary File S1. [file 41598_2024_63226_MOESM6_ESM.zip › RoPodv5/Sliced file - ready to print/RoPod5-2_Supl-preview_5.png]

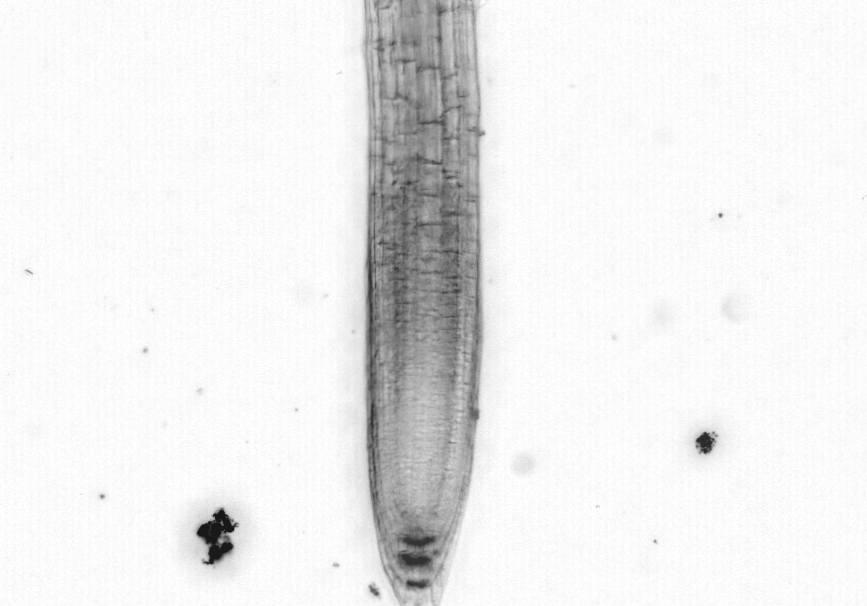

Supplement: Supplementary file 8 — Supplementary File S9. [file 41598_2024_63226_MOESM8_ESM.zip › image and results example/20220322_Col_sucrose_Vert_RoPod5_B2_R10_EDF-RW-GPU_Bckg-Stitch-reg_2.tif]

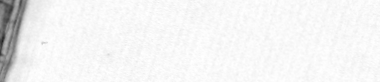

Supplement: Supplementary file 8 — Supplementary File S9. [file 41598_2024_63226_MOESM8_ESM.zip › image and results example/results/20220322_Col_sucrose_Vert_RoPod5_B2_R10_EDF-RW-GPU_Bckg-Stitch-reg_2_roi_01_Straigthen-BF.tif]

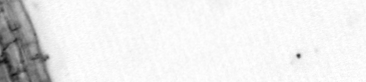

Supplement: Supplementary file 8 — Supplementary File S9. [file 41598_2024_63226_MOESM8_ESM.zip › image and results example/results/20220322_Col_sucrose_Vert_RoPod5_B2_R10_EDF-RW-GPU_Bckg-Stitch-reg_2_roi_02_Straigthen-BF.tif]

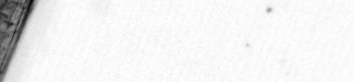

Supplement: Supplementary file 8 — Supplementary File S9. [file 41598_2024_63226_MOESM8_ESM.zip › image and results example/results/20220322_Col_sucrose_Vert_RoPod5_B2_R10_EDF-RW-GPU_Bckg-Stitch-reg_2_roi_03_Straigthen-BF.tif]

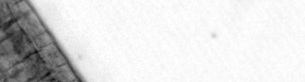

Supplement: Supplementary file 8 — Supplementary File S9. [file 41598_2024_63226_MOESM8_ESM.zip › image and results example/results/20220322_Col_sucrose_Vert_RoPod5_B2_R10_EDF-RW-GPU_Bckg-Stitch-reg_2_roi_04_Straigthen-BF.tif]

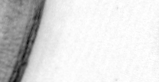

Supplement: Supplementary file 8 — Supplementary File S9. [file 41598_2024_63226_MOESM8_ESM.zip › image and results example/results/20220322_Col_sucrose_Vert_RoPod5_B2_R10_EDF-RW-GPU_Bckg-Stitch-reg_2_roi_05_Straigthen-BF.tif]
